# Surface Properties of Saponin—Chitosan Mixtures

**DOI:** 10.3390/molecules27217505

**Published:** 2022-11-03

**Authors:** Marcel Krzan, Natalia García Rey, Ewelina Jarek, Agnieszka Czakaj, Eva Santini, Francesca Ravera, Libero Liggieri, Piotr Warszynski, Björn Braunschweig

**Affiliations:** 1 Jerzy Haber Institute of Catalysis and Surface Chemistry, Polish Academy of Sciences, ul. Niezapominajek 8, 30-239 Krakow, Poland; 2 Institute of Physical Chemistry and Center for Soft Nanoscience, Westfälische Wilhelms-Universität Münster, Corrensstraße 28/30, 48149 Münster, Germany; 3 Institute of Condensed Matter and Technologies for Energy, Consiglio Nazionale delle Ricerche, Via Marini 6, 16149 Genova, Italy

**Keywords:** saponin, chitosan, saponin—chitosan interactions, bio-compatible systems, biodegradable surfactants

## Abstract

The surface properties of saponin and saponin-chitosan mixtures were analysed as a function of their bulk mixing ratio using vibrational sum-frequency generation (SFG), surface tensiometry and dilational rheology measurements. Our experiments show that saponin-chitosan mixtures present some remarkable properties, such as a strong amphiphilicity of the saponin and high dilational viscoelasticity. We believe this points to the presence of chitosan in the adsorption layer, despite its complete lack of surface activity. We explain this phenomenon by electrostatic interactions between the saponin as an anionic surfactant and chitosan as a polycation, leading to surface-active saponin-chitosan complexes and aggregates. Analysing the SFG intensity of the O-H stretching bands from interfacial water molecules, we found that in the case of pH 3.4 for a mixture consisting of 0.1 g/L saponin and 0.001 g/L chitosan, the adsorption layer was electrically neutral. This conclusion from SFG spectra is corroborated by results from surface tensiometry showing a significant reduction in surface tension and effects on the dilational surface elasticity strictly at saponin/chitosan ratios, where SFG spectra indicate zero net charge at the air–water interface.

## 1. Introduction

Using environmentally friendly resources to manufacture surfactants is desirable to reduce the environmental impact of production and their use in cosmetic, pharmaceutical and food technologies. In the last half-century, the development of biodegradable surfactants for biomedical applications has advanced significantly [1,2,3]. Saponin solutions and saponin-chitosan mixtures are natural and biodegradable compounds with surface-active and antimicrobial properties [4,5,6,7,8]. Saponins are a group of plant secondary metabolites that exhibit surface-active properties. Chemically, saponins contain glycosylated steroids, triterpenoids and steroid alkaloids. Currently, saponins are used as foamers and emulsifiers in beer and soft drinks [9,10], as solubilising agents for vitamins [11] and minerals [12], as surfactants in cosmetics [9,13,14] or food and feed additives [13] and also for the reduction of lipids administration [15].

Chitosan is a natural biopolymer that is contained in the shells of insects, marine crustaceans and fungi’s cell walls. As a non-toxic, biodegradable carbohydrate polymer, chitosan is used for biomedical and food applications [16,17].

Our previous works evidenced that saponin presents amphiphilic properties relevant to its utilisation as a foam stabiliser or emulsifier [4,18]. Foam stability was well correlated with dilatational surface elasticity, which describes the mechanical response of the interface to small surface area perturbation. This is usually determined by measuring the dynamic surface tension that results from low amplitude harmonic variations of the surface area. Shear interfacial elasticity, measured by a different technique, defines the strength of intermolecular interactions at the interface, which influences thin liquid film drainage and is relevant for foam stability [19]. The viscoelastic response of saponins at the liquid–air interface varies depending on their chemical structure. Triterpenoid saponins show an extremely high viscoelastic response as well as high dilatational and shear moduli, while steroid-type saponins present a more liquid-like response to shear stress [20].

We found that the surface activity of triterpenoid saponin can be further increased in the presence of acetic acid, which is caused by the protonation of carboxylic groups at low pH. This induces a transition from an ionic to a non-ionic form of the surfactant [4]. In addition, we have also reported that adding chitosan to the saponin solutions increases the viscoelastic effects on the interfacial and bulk properties, with a significant enhancement of the liquid content and long-time stability of the respective aqueous foams. However, the nature of those interactions and their strength could not be evaluated based on the previously obtained experimental data.

In our previous work [4], in fact, due to the lack of surface activity of chitosan, we assumed its presence mainly in the liquid film, and we hypothesised the formation of amphiphilic saponin—chitosan complexes transferring into the interfacial layer without direct proof. Moreover, we worked there with relatively high concentrations of chitosan (above 0.1 g/L) and in pH 2.85 (1 wt% of acetic acid), where the saponin (an anionic surfactant) is mostly undissociated.

In the present study, we have studied the saponin-chitosan systems at a higher pH for a range of concentrations of chitosan from 0.0001 to 0.01 g/L. At this pH of 3.4, the saponin is more dissociated. Very low concentrations of chitosan were not tested in the previous work, where its minimum concentration was 0.1 g/L.

We have now incorporated complementary information from in situ nonlinear optical spectroscopy–vibrational sum-frequency generation spectroscopy (SFG), which allows direct identification and analysis of compounds adsorbed in the interfacial layer [21,22]. This analysis targets specific functional groups such as -methylene, -methyl, -hydroxy groups and their molecular structure at the interface, while the interfacial charge can be qualitatively addressed by SFG spectroscopy using O-H stretching modes from interfacial water molecules. Using SFG spectroscopy, we aimed to resolve the composition and structure of the adsorption layer on a molecular level, allowing us to provide new information on chitosan-saponin mixtures at the air–water interface. Furthermore, we show that chitosan was contained in the adsorption layer, despite the lack of its surface-active properties and discuss a hypothesis explaining this phenomenon. We also performed the surface tension and surface dilational rheology test in the same new saponin-chitosan concentrations.

## 2. Experimental Results

**Figure 1:**
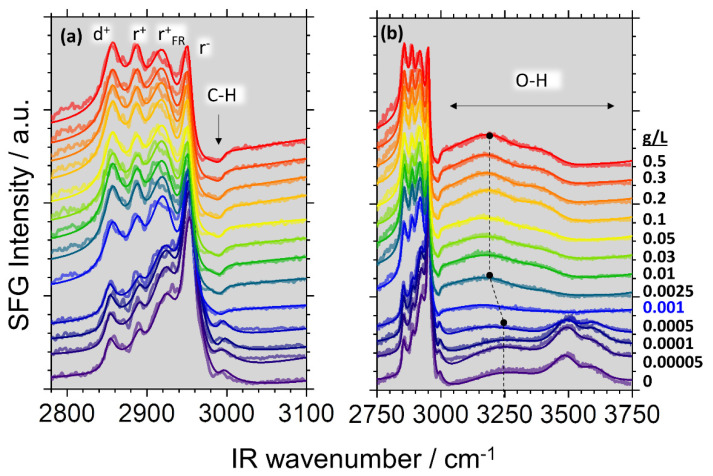
Vibrational SFG spectra of air–water interfaces modified by saponin–chitosan mixtures as a function of the chitosan concentration, which was as indicated on the right axes of the panel (**b**). Note that the saponin concentration in the aqueous solution was fixed at 0.1 g/L, while the chitosan concentration was varied. The solution contained 0.1 wt% acetic acid in order to adjust the pH value to 3.4 for all chitosan concentrations. Spectra were offset for better visual inspection. Panel (**a**) shows a magnified view of the SFG spectra that concentrate on the details of C-H modes from interfacial molecules. Bands assignments to CH_2_ (d^+^) and CH_3_ (r^+^), the Fermi resonance r^+^_FR_, the CH_3_ asymmetric stretch (r^−^) and a C-H stretch (C-H) are labelled in (**a**) and also discussed in the main text. Panel (**b**) overviews spectra that show both C-H and O-H modes from interfacial molecules, such as the broad O-H stretching modes which are indicated. The dotted line highlights the red shift of the OH frequency as the concentration of chitosan increases. (See details in the main text).

Figure 1 shows a series of SFG spectra where we have addressed C-H and O-H vibrations from the adsorbate layer of saponin-chitosan mixtures at the air–water interface. For that, the saponin concentration was fixed at 0.1 g/L while the chitosan concentration was varied from 0 to 0.5 g/L. The solutions were mixed in water with 0.1% acetic acid, and the pH was adjusted to 3.4 for all mixtures. Figure 1a shows a detailed view of the C-H region, where the band assignments are also indicated in the figure, and the symmetric stretching modes of methylene (d^+^) and methyl (r^+^) groups, the fermi resonance of the methyl (r^+^_FR_) and the methyl asymmetric stretch (r^−^) give rise to vibrational bands at frequencies of ~2855, ~2890, ~2927 and ~2952 cm^−1^, respectively [23,24,25,26,27,28,29]. Chitosan shows an additional C-H band at ~2994 cm^−1^, which is observed in the SFG spectra of the pristine air–saponin interface [22,30,31,32,33,34,35] (see also our results, Figure S2a). Acetic acid shows no SFG spectra in studied concentration 0.1 g/L [36] (see also Figure S3). Additional broad vibrational bands due to O-H stretching modes from hydrogen-bonded interfacial molecules are found between 3000 and 3800 cm^−1^. These bands are very sensitive to the structure of the contributing water molecules and to the strength of the hydrogen bonds within the network of interfacial water molecules [37,38]. Close inspection of Figure 1b shows that the shape and intensity of the O-H stretching bands change drastically as a function of the chitosan concentration. There are three distinct water structures at the saponin–chitosan interface depending on the frequency of the O-H stretching band of the water molecules around the interface.

(i) At high chitosan concentrations, the SFG spectra in Figure 1b exhibit two main features with O-H stretching bands centred at ~3200 and ~3450 cm^−1^. The low-frequency band is attributed to more tetrahedrally coordinated water molecules, while the high-frequency band is due O-H groups in more disordered or less tetrahedrally coordinated environments [39,40]. The dip between these two features has been attributed to the intermolecular coupling between the O-H stretch and the bending overtone by Sovago et al. [41].

(ii) The high-frequency part of the SFG spectrum shows for chitosan concentrations lower than 0.001 g/L (Figure 1b) clearly O-H bands at 3500 cm^−1^ and a broad shoulder at 3600 cm^−1^, which both dominate the SFG spectra for these low chitosan concentrations (<0.001 g/L). Similar bands have been observed at low pH for β-escin as another saponin at the air–water interface. For β-escin, the vibrational bands at 3500–3600 cm^−1^ were attributed to O-H stretching modes at the hydrophilic oligosaccharide section that originate presumably from the protonated carboxylic acid group, which is only present at low pH [21]. In fact, O-H bands at frequencies > 3500 cm^−1^ have been frequently assigned to weakly hydrogen-bonded water molecules [42,43,44] close to carbonyl groups at lipid-covered surfaces. However, as the chitosan concentration increases, O-H bands from the oligosaccharide section (glycone) of the saponin decrease substantially in intensity until they are negligible at 0.001 g/L chitosan.

(iii) In addition, we attribute the weaker band at about ~3600 cm^−1^ to interfacial water molecules close to the hydrophobic parts of the saponin–chitosan complexes at the air–water interface. This assignment is corroborated by the work of Modal et al., who studied a zwitterionic lipid at the air–water interface [45], were at the high frequency side of the O-H spectrum showed a broad O-H feature at about 3620 cm^−1^, which the authors attributed to water molecules close to the hydrophobic region of the lipid molecules. A notion that is also supported by several other experimental studies as well as by molecular dynamics MD simulations [43,46,47].

**Figure 2:**
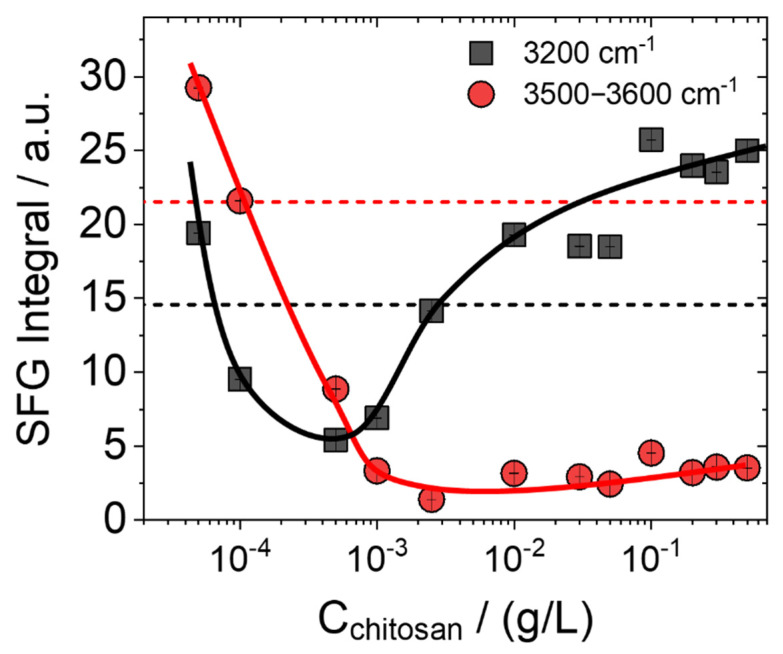
Integrated SFG intensities of O-H stretching bands (3055–3355 cm^−1^) related to the hydrogen-bonded interfacial water molecules (black squares), which is also dependent on the net charging state of the interface, as discussed in the main text. Integrated SFG intensities (3393–3695 cm^−1^) from O-H groups at the oligosaccharide glycone of the saponin (red circles) as well as from water molecules close to the hydrophobic parts of the chitosan/saponin mixtures at the interface are also shown as a function of the chitosan concentration. Red and black dashed lines show the SFG intensities of both contributions to the SFG spectra (bands at 3200 and ~3550 cm^−1^). Solid lines guide the eye.

In order to get more quantitative information on the change of O-H intensities, we have analysed the SFG spectra in Figure 1 in more detail and plotted in Figure 2 the integrated intensity (see details section and figure caption) of the 3200 and ~3500/3600 cm^−1^ bands in dependence on the chitosan concentration. Clearly, a minimum value at 0.001 g/L chitosan is established.

**Figure 3:**
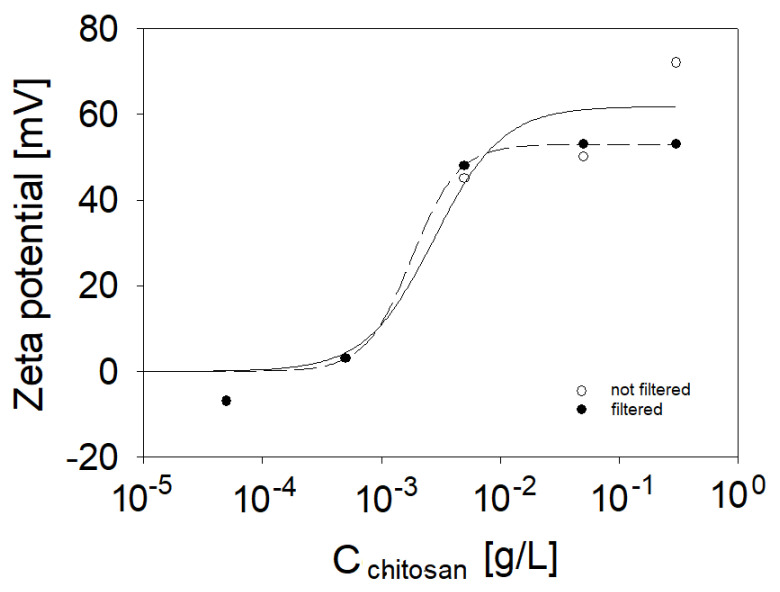
Zeta-potential for saponin or saponin–chitosan aggregates in mixtures. Fixed saponin concentration of 0.1 g/L in the presence of 1 wt% acetic acid. Chitosan concentrations varied in a range from 0.0005 to 0.5 g/L. Solid and dashed lines guide the eye (solid line show the not filtered aggregates, dashed—filtered).

Next, we will address the apparent decrease and subsequent increase in the intensity of the low-frequency O-H band, e.g., at 3200 cm^−1^ from hydrogen-bonded interfacial water (Figure 2). For that, recall that the SFG intensity can depend on both second- and third-order contributions, where the latter is related to the double-layer potential at the interface and can be, thus, used to study the charging state at an interface qualitatively (see Equation (3) below). In Figure 3, we show that bulk measurements of the zeta-potential reveal a charge reversal of the saponin–chitosan aggregates formed in the bulk solutions. For further discussion, we point out that the chitosan macromolecules are polycationic at low pH because their polysaccharide chain contains amine groups that can be protonated and thus positively charged when the pH is low enough.

In contrast, the saponins at low pH are either uncharged or slightly negatively charged. Indeed, our bulk zeta-potential measurements show a negative zeta-potential of saponin aggregates in the absence of chitosan (−16.9 mV at a saponin concentration of 0.1 g/L in an aqueous solution). The mixture of saponin with 1 wt% acetic acid shows a zeta-potential of ca. −6 mV only, which indicates that the addition of acetic acid can partially neutralize the negative charge of saponin particles.

The addition of even a small amount of chitosan caused the charge reversal. The mixture presented a positive zeta-potential charge up to approximately +70 mV at the highest chitosan concentration of 0.5 g/L (see Figure 3). These results confirm that in the studied range of chitosan concentration, there is a change in the surface potential of particles in saponin solutions. Chitosan-saponin mixtures are, at low pH, conceptually similar to the well-studied mixtures of oppositely charged polyelectrolyte and surfactants (e.g., mixtures of polycations with anionic surfactants). In similar systems, the charge reversal was observed in bulk as well as at the air–water interface with predominant spread polyelectrolyte-surfactant aggregates [48,49,50,51]. For that reason, and since the dependence of O-H signals in SFG spectra provides information on the interfacial charging state (Equation (3)), we assign the changes in O-H intensity to changes in the net interfacial charge. To corroborate this assignment, we discuss the shape of the C-H band (Figure 1) at about 2994 cm^−1^ that is indicated by the arrow in Figure 1a. At chitosan concentrations <0.001 g/L, this band appears as a positive going or peak-like feature, while at concentrations >0.001 g/L, the spectral shape of this band changes to a shallow dip-like feature. We recall that the appearance of vibrational bands in SFG spectroscopy depends on the molecular orientation (see description of vibrational SFG spectroscopy in the details section below). Consequently, the changes in the shape of the vibrational band centred at 2994 cm^−1^, can be attributed to a changed net orientation of the interfacial water molecules. Indeed, fitting of the SFG spectra (see Supporting Information) at the lowest and highest chitosan concentration shows that the relative phase of the water band at 3200 cm^−1^ and the band at 2994 cm^−1^ changes by *π*. As the phase of a vibrational band in SFG spectroscopy is related to the net molecular orientation, this further corroborates our conclusions on a charge reversal at the interface. Note that similar changes of C-H bands in SFG spectra caused by a flip in the water net orientation have been previously reported for proteins as well as for polyelectrolyte-surfactant mixtures when the interfacial point of zero charge was crossed, e.g., by adjusting the pH, the mixing ratio or the polyelectrolyte concentration [34,49,52]. Furthermore, it is also interesting to discuss the molecular origin of the decrease in intensity of the high-frequency bands at 3500 and 3600 cm^−1^, which are neglectable when the chitosan concentration is >0.001 g/L. A similar reduction was observed for β-escin (another saponin)—when the pH was increased [21]. This was attributed to carboxylate groups’ protonation at the glycone of β-escin. Adsorption of positively charged chitosan-saponin aggregates and subsequent formation of an electric double layer at the air–water interface may reduce the hydronium concentration at the interface and, thus, shift the local pH to a higher value (see also Sthoer and Tyrode [53]). The change in local pH may also cause some deprotonation of the saponins’ carboxylic acid groups, which can help stabilize the saponin–chitosan aggregates at the air–water interface. In addition, we observe a red-shift of the O-H stretching band of interfacial water from 3250 cm^−1^ to 3200 cm^−1^ at chitosan concentrations higher than 0.001 g/L. This frequency shift may be associated to stronger intermolecular coupling of the O-H bands of the water molecules at the interface as the saponin-chitosan aggregates become more closely packed at the air–water interface [37]. Moreover, the N-H stretch (~3300 cm^−1^) from amine groups at the polysaccharide chain of the chitosan may also contribute to the stronger coupling to the O-H stretching modes [54].

**Figure 4:**
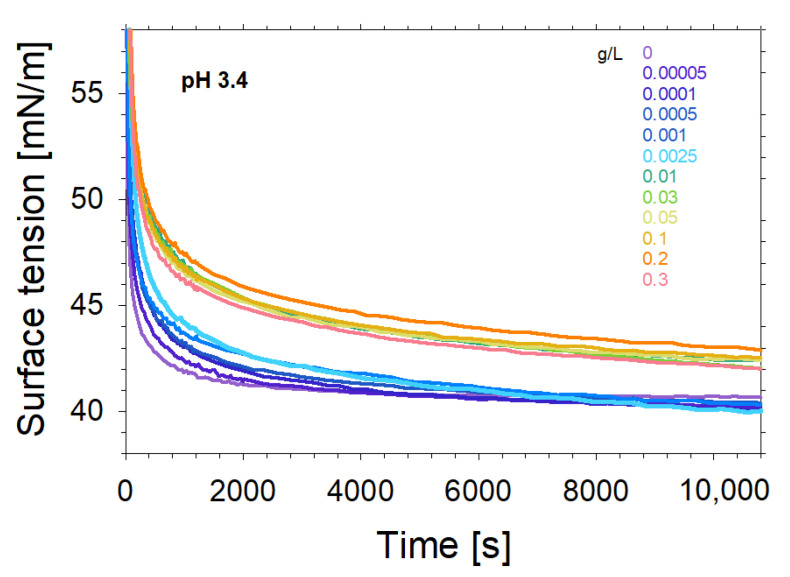
The dynamic surface tension of saponin solutions and saponin–chitosan mixtures in the presence of 0.1 wt% acetic acid and a solution pH of 3.4. The Saponin concentration was fixed at 0.1 g/L, while the chitosan concentration was varied from 0.0005 to 0.3 g/L as indicated in the figure.

**Figure 5:**
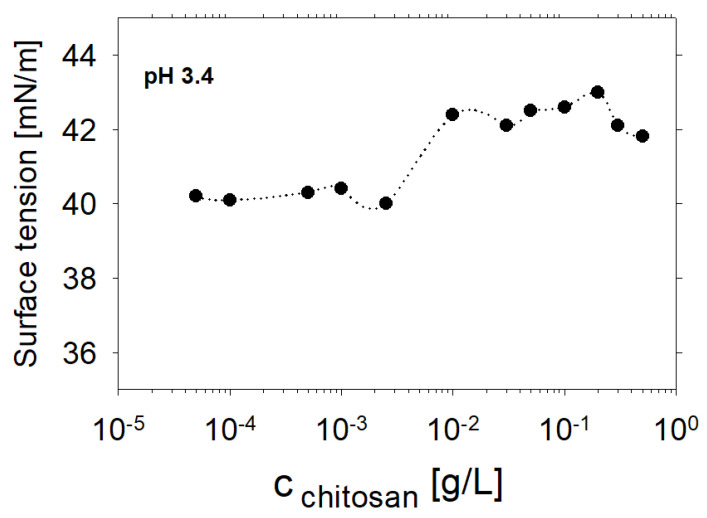
Equilibrium surface tensions of saponin solutions and saponin–chitosan mixtures in the presence of 0.1 wt% acetic acid for pH 3.4 that were measured after 3 h (>10,800 s) of adsorption. The saponin concentration was fixed to 0.1 g/L while we varied the chitosan from 0.0005 to 0.3 g/L.

As next we concentrated on the adsorption properties and dilational rheology of saponin-chitosan mixtures. Figure 3 presents the dynamic surface tension of saponin–chitosan mixtures with acetic acid at the air–water interface, which is dominated by adsorption kinetics. Clearly, after an adsorption time of 3 h, the interfaces modified by saponin–chitosan mixtures are close to an equilibrium state for all studied mixing ratios. However, despite the lack of chitosan’s surface activity, even trace amounts of chitosan can noticeably influence the state of adsorption at the air–water interface. That is clearly evidenced by the changes in adsorption kinetics, which slow down as the chitosan concentration increases. In particular, the dynamic surface tension at early adsorption times reports on the changed kinetics as it systematically shifts to longer adsorption times when the chitosan concentration is increased. However, also at close to equilibrium conditions, the surface tension changed with concentration. Therefore, we compared the surface tension, for example, at the end of the kinetic series at 10,800 s (see Figure 4). In Figure 5, we plot those surface tensions as a function of chitosan concentration.

At concentrations below the limit of 0.0025 g/L of chitosan, we observed a lower equilibrium surface tension of about 40 mN/m, which was nearly independent of the polycation concentration. On the contrary, when exceeding a concentration of 0.0025 g/L of chitosan, the equilibrium surface tension increased to a value of about 42 mN/m. The observed equilibrium values have a higher experimental uncertainty than in the case of lower concentrations, but the difference between both levels is a few times over the experimental apparatus error (~0.2 mN/m). The observed effect corresponds to the charging of the interfacial layer upon the adsorption of chitosan, as discussed above. Indeed, the chitosan concentration where the noticeable jump in surface tension occurs is consistent with the results of the SFG measurements, which show a minimum in O-H intensity and, thus, surface charging.

**Figure 6:**
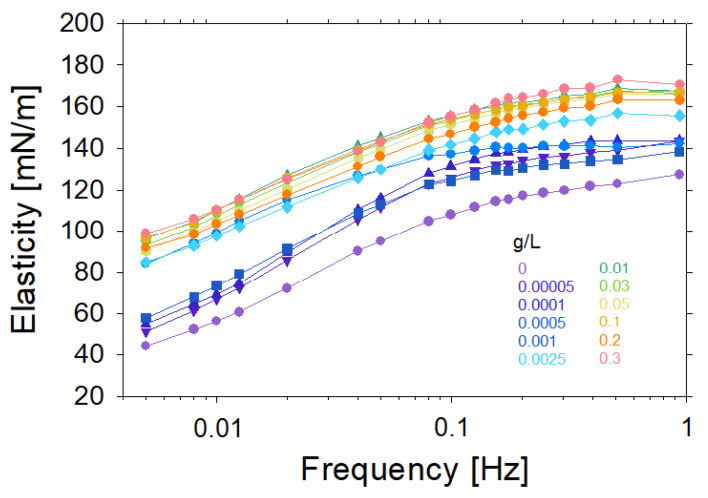
Dilational viscoelasticity versus frequency measured by the oscillating drop method in the presence of 0.1 wt% acetic acid and a solution pH of 3.4. The saponin concentration was fixed to 0.1 g/L, while the chitosan concentration was varied from 0.0005 to 0.3 g/L as indicated in the figure.

As seen in Figure 6, the dilational interfacial elasticity rises in the presence of chitosan for concentrations up to 0.3 g/L. The analysis of changes in surface elasticity concerning the oscillation frequency for different chitosan contents allows us to easily distinguish the transition from low chitosan concentrations, where the surface, according to our SFG observations, was negatively charged, to the conditions of high concentration of added polycation (chitosan), where the surface charge is already completely neutralized (Figure 1 and Figure 2 and their discussion). As shown in Figure 6, the elasticity also confirms the transition point at a chitosan concentration of 0.001 g/L.

**Figure 7:**
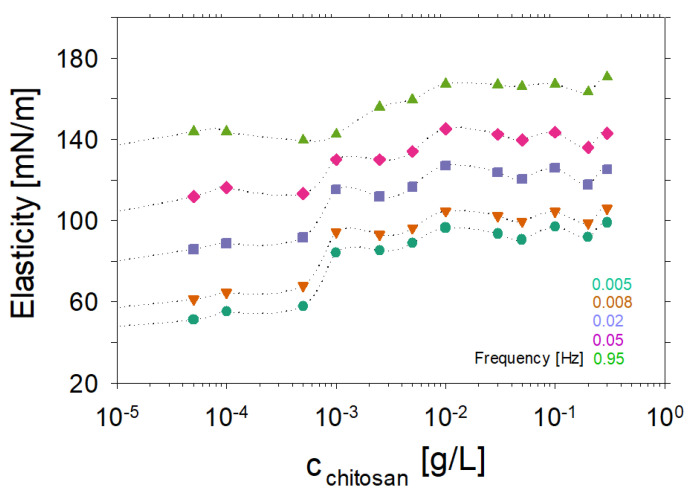
Surface dilational elasticity results as a function of chitosan concentration in aqueous mixtures with saponin 0.1 g/L and acetic acid 0.1 wt% (pH 3.4). Plots for five various oscillation frequencies, which were 0.005, 0.008, 0.02, 0.05 and 0.95 Hz, as indicated in the figure, are shown.

Figure 7 shows the surface elasticity as a function of the chitosan concentration in the mixtures with saponin. Data are presented for five different frequency oscillations. As can be seen, there is a significant difference in the surface elasticities between low and high chitosan solutions. The boundary between them is the concentration of 0.001 g/L where the net charge at the air–water interface modified by chitosan-saponin mixtures is negligible.

## 3. Discussion and Summary

In this work, we have studied the effect of the chitosan concentration on the interfacial properties of mixtures with a saponin biosurfactant by combining vibrational SFG spectroscopy with surface tension and dilational elasticity measurements. These measurements were accompanied by determining the zeta-potential from saponin–chitosan aggregates in the bulk solution. At a chitosan concentration of 0.001 g/L, a minimum in O-H intensity from interfacial water molecules is observed and attributed to a charge reversal and overcharging of chitosan-saponin complexes at the air–water interface. Additionally, for lower chitosan concentrations, a clear feature in the vibrational SFG spectra coming due to hydroxyl groups from the saponin glycone is observed, whereas at higher chitosan concentrations, SFG spectra are dominated by broad O-H stretching modes of interfacial water molecules. We have used the relative phase between the O-H and the C-H band and thus the spectral shape of the C-H band that is closer in frequency to the O-H bands to qualitatively determine the flip in the net orientational of interfacial water when the net charging state is reversed at the air–water interface. The results from SFG spectroscopy showed that chitosan is part of the surface adsorption layer, despite the lack of its surface-active properties. The surface activity and dilational elasticity experiments confirmed the effect indicated by SFG spectroscopy. We provide experiments that clearly show that the surface activity of the saponin–chitosan mixtures decreases with increasing concentration of chitosan. The surface elasticity of the analysed system also changes with the mixing ratio of both components.

We propose that the observed phenomenon results from electrostatic interactions between negatively charged particles of anionic saponin and the positively charged polycation chains of chitosan. We provide information on chitosan as a polycation itself has poor surface activity, but it can develop cooperative effects with a saponin which leads to enhanced surface properties with more surface-active complexes or aggregates that are driven by the interaction with the saponin. This phenomenon can be used in the future formulation of cosmetic and therapeutic foams and emulsions to deliver chitosan to the surface layer. Chitosan, in the form of such a complex, should easily penetrate the skin’s pores, enhancing its healing effect. At this point, it should also be noted that the saponin–chitosan complexes show attractive surface-active properties that can be used to formulate new emulsions and cosmetic foams.

## 4. Materials and Methods

### 4.1. Materials

Saponin extracted from *Quillaja Saponaria* bark in the form of white powder was purchased from VWR International (Saponin, Reagent Grade catalogue number VWRV0163, LOT 1666C426), Amresco life science, reagent grade) and was used as received. The molecular formula and molecular weight are not well defined for this product. However, as in many studies on saponins or mixtures of saponins, [20,55,56,57] we assume an average molecular weight of 1650 g·mol^−1^.

Chitosan (β-(1-4) linked 2-amino-2-deoxy-D-glucose) low molecular weight (50–190 kDa) was purchased from NANOSHEL as high-density powder with a purity degree of 99.9% and used as received. To prepare the solutions, we dissolved chitosan powder in 1 wt% acetic acid solution under an overnight continuous magnetic bar stirring.

Acetic acid was purchased from Sigma-Aldrich with a purity higher than 99.7% and was used without further purification.

The ultrapure water used in this study was obtained from a Millipore (Elix + Milli-Q) purification system, warranting a resistivity higher than 18 MΩ∙cm and stable surface tension of about 72.4 mN/m at 22 °C. All the glass and Teflon laboratory materials utilised in the samples preparation and measurements were (i) initially stored and cleaned with Mucasol universal laboratory detergent, (ii) cleaned with potassium dichromate solution in sulphuric acid and then (iii) rinsed with Milli-Q water.

For SFG spectroscopy, the needed glassware was cleaned in a mixture of 98% concentrated sulfuric acid (Carl Roth, Karlsruhe, Germany) with NOCHROMIX (Godax Labs, Cabin John, MD, USA) for at least 12 h. Subsequently, it was thoroughly rinsed with ultrapure water from a Millipore Reference A+ purification system and dried in a stream of 99.999% nitrogen gas (Westfalen, Germany). For SFG, 3.3 mL of the saponin–chitosan solutions were loaded into an acid-cleaned Petri dish and were allowed to equilibrate for 30 min before data acquisition. All experiments were carried out at 297 K room temperature.

### 4.2. Sample Preparation

The stock solution of saponin was prepared by dissolving it in water and leaving it for 8 h to protonate all biosurfactant molecules. Meanwhile, to fully dissolve chitosan, its solution was prepared in the presence of 1 wt% acetic acid. The chitosan stock solution was mixed on a magnetic stirrer for over 8 h until the biopolymer was fully dissolved. Both stock solutions were prepared not earlier than 48 h before experiments.

The stock solutions of saponin and chitosan were filtered to avoid the presence of large aggregates and their spreading to the air–water interface. However, according to SFG spectroscopy, filtering of the samples had little effect on the air–water interface as there were hardly any noticeable differences between SFG spectra of filtered and unfiltered solutions (Figure S1, Supplementary Materials). Furthermore, zeta-potential experiments also confirmed that the effect of filtering on obtaining results is negligible (see Figure 3).

Mixtures of saponin and chitosan were prepared just before the experiment. Then, an appropriate amount of acetic acid was added to each of them to have a pH equal to 3.4.

### 4.3. Methods

#### 4.3.1. Sum-Frequency Generation Spectroscopy

Vibrational sum-frequency generation (SFG) is a second-order nonlinear spectroscopy with unique selectivity and sensitivity to study interfaces on a molecular level. SFG has been applied to study a range of interfaces such as gas–solid [58,59,60], liquid–solid [61,62,63] or gas–liquid [35,54,64,65], becoming the only method that can provide interface-selective vibrational spectra on the water structure at aqueous interfaces. [40,66] The SFG intensity $I_{SFG}$ is directly proportional to the absolute square of the sum-frequency polarization $P_{SFG}^{\left(2\right)}$,

(1) $$I_{SFG}\propto {\left|P_{SFG}^{\left(2\right)}\right|}^{2}\propto {\left|\chi^{\left(2\right)}\right|}^{2}I_{IR}I_{vis}$$

$P_{SFG}^{\left(2\right)}$ is induced by two incident beams, a frequency-tunable broadband IR beam ($\omega_{IR})$ and a visible beam at a fixed frequency narrowband ($\omega_{vis}$), which has a narrow bandwidth. The overlap of the two beams in time and space gives rise to a third beam with the sum-frequency ($\omega_{SFG}=\omega_{IR}+\omega_{vis}$) of the two fundamental beams. Within the dipole approximation, the second-order nonlinear susceptibility $\chi^{\left(2\right)}$ is only non-zero when the inversion of symmetry is broken, which is the case for interfaces [67]. In fact, this makes SFG a powerful tool to study surfaces and interfaces as it is highly interface specific because bulk signals in materials with inversion symmetry like isotropic liquids and gases are negligible. Interfaces, however, break the prevailing bulk symmetry and allow for SFG signals that can be used to study the interfacial molecular structure. The second-order electric susceptibility $\chi^{\left(2\right)}$ depends on a nonresonant (NR) contribution $\;\chi_{NR}^{\left(2\right)}$ and a resonant contribution $\;\chi_{R}^{\left(2\right)}$,

(2) $$\;\chi_{R}^{\left(2\right)}=\underset{k}{\sum }\frac{A_{k}}{\omega -\omega_{k}+i\gamma_{k}}$$

where $A_{k}$, $\omega_{k}$ and $\gamma_{k}$ are the amplitude, resonance frequency and linewidth of the qth vibrational mode. The amplitude $A_{k}\propto N\langle \beta_{q}^{\left(2\right)}\rangle$ is a function of the number density of interfacial and the orientational average of the molecular hyperpolarizability $\beta_{q}^{\left(2\right)}$. Consequently, the shape of vibrational bands in homodyned SFG spectra depends on the orientation of interfacial molecules. The $\chi_{NR}^{\left(2\right)}$ contribution is dominated by electronic excitations at the interface and is, in case of optically transparent systems, often negligible while $\chi_{R}^{\left(2\right)}$ originates from molecular vibrations of interface-adsorbed molecules. These can be resonantly excited by the impinging IR beam and give rise to resonantly enhanced SFG signals [35,68,69]. In addition, an additional third-order contribution $\;\chi_{R,EDL}^{\left(3\right)}$ needs to be considered at charged interfaces, which can be related to a contribution from the electric double-layer that breaks the symmetry at the interface and which is proportional to the double-layer potential $\phi_{0}\;$ [32,70,71,72,73]. Clearly, this dependence offers the possibility to interrogate the interfacial charging state at least on a qualitative level,

(3) $$I_{SFG}\propto {\left|\chi_{NR}^{\left(2\right)}+\chi_{R}^{\left(2\right)}+\frac{\kappa }{\kappa -i\Delta k_{z}}\chi^{\left(3\right)}\phi_{0}\right|}^{2}$$

Here κ is the inverse Debye length and $\Delta k_{z}$ the wave vector mismatch between IR and vis beam.

Although the information of the absolute orientation of the surface molecules is lost in homodyne-detected vibrational SFG [67,74,75], the interference between the vibrational modes can also reveal a charge reversal or a significant change on the charging state of the interface. This is monitored by a relative phase change between modes or the nonresonant background [23,76]. Previous studies have shown how the phase of the O-H bands causes constructive or destructive interference if the overall orientation of the O-H molecules varies, e.g., when a charge reversal at the interface occurs.

The integration intervals for the O-H band intensities, whose results are shown in Figure 2, are for the O-H peak at 3200 cm^−1^ {3055–3353 cm^−1^} and for the O-H peak at 3600 cm^−1^ {3393–3695 cm^−1^}.

The set-up for our home-built SFG spectrometer is described in detail elsewhere [71]. SFG spectra in the frequency region from 2700 to 3800 cm^−1^ were taken by tuning the IR centre frequency in four steps with a spectral width of >300 cm^−1^. The maximum pulse energy of the broadband femtosecond IR beam was set to 15 µJ to avoid radiation damage of the samples. The acquisition time for each centre IR frequency was 180 s, resulting in a total acquisition time of 15 min per SFG spectrum. The polarizations of the SFG, VIS, and IR beams were set to SSP, respectively. The SFG spectra were normalized with the nonresonant contribution from an Au thin film on a Si wafer, which was cleaned in an air plasma before each experiment.

#### 4.3.2. Surface Activity and Dilational Surface Elasticity

Surface tension and dilational viscoelasticity measurements were performed using a drop shape tensiometer Sinterface PAT1M (Sinterface Technologies e.K., Berlin, Germany). Details on these techniques and our experimental procedure are reported elsewhere [4,77]. For the present study, the tensiometer was used in the pendant drop configuration. The interfacial tension was acquired by maintaining a drop volume of about 11 mm^3^ and a constant surface area after the rapid creation of a drop at the tip of a hydrophobic stainless-steel capillary. This way, the adsorption kinetics were monitored starting from a nearly “fresh” surface.

The dilational viscoelasticity (*E*) is the dynamic response of the interfacial tension, γ, to extensional perturbations of the surface area, A. The surface dilational elasticity measurements as a function of frequency were obtained using the oscillating drop method, which was started after achieving an adsorption equilibrium (t > 3 h). The amplitude of the sinusoidal area perturbations was such to ensure the linear response of the interfacial layer and, in a frequency range from 0.005 to 0.2 Hz, to maintain a Laplacian profile of the pendant drop during oscillation. For small amplitude harmonic perturbations, *E* is a frequency-dependent complex quantity, defined as,

(4) $$E=\frac{\Delta \gamma }{\Delta A/A_{0}}e^{i\phi }$$

where Δγ and Δ*A* are the amplitudes of the oscillating surface tension and surface area, respectively, while *A*_0_ is the reference area, and *ϕ* is the phase shift between the oscillating surface tension and surface area. The accuracy of experiments was better than 0.2 mN/m, which was confirmed by repeated tests with the same concentrations of saponin–acetic acid–chitosan mixtures.

#### 4.3.3. Zeta-Potential

The zeta-potential was measured using laser doppler electrophoresis and dynamic light scattering (Zetasizer Nano Series, Malvern Panalytical, Malvern, UK) with disposable measurement cells (DTS 1065, Malvern). Zeta-potentials were calculated from the electrophoretic mobility of particles using the Smoluchowski model. Each value was obtained as an average from three consecutive measurements with 20 runs.

## Data Availability

The data presented in this study are freely available from the authors upon a reasonable request.

## Supplementary Materials

The following supporting information can be downloaded at: https://www.mdpi.com/article/10.3390/molecules27217505/s1, Including the SFG spectra of filtered and unfiltered pristine saponin air–water interface, pristine saponin for different concentrations and neat chitosan at the air–water interface. Figure S1. Comparison of SFG spectra of air-water interfaces from filtered and unfiltered saponin solutions with a concentration of 0.1 g/L. The polarization of the SFG, VIS and IR beam is SSP. For the filtered sample the stock solution of saponin was filtered with a 0.1 µm filter; Figure S2. SFG spectra of the pristine saponin air water interface. (a) study of the effect of the saponin concentration, pH 5.2 and (b) study of the addition of 1% acetic acid to the solutions (pH 2.85). The polarization of the SFG, VIS and IR beam was SSP. The pH of solutions with 1% of acetic acid becomes more acidic, pH 2.85, and the OH bands are highly affected by the double layer contribution, decreasing in SFG intensity due to the decrease in the net charge at the interface. The C-H bands also are affected by the tail of the broad OH stretching bands, as the SFG spectra are dependent on the interference between the bands and the relative phases. See references for detailed explanation; Figure S3. Comparison of SFG spectra of 0.5 g/L chitosan air-water interface in 96% or 1% of acetic acid solution. The polarization of the SFG, VIS and IR beams is SSP, respectively; Figure S4. Comparison of the fit curves (red lines) with the experimentally determined vibrational SFG spectra (black lines) of air-water interfaces modified by saponin-chitosan mixtures with chitosan concentrations of 0 and 0.5 g/L as indicated in the figure. Note that the saponin concentration in the aqueous solution was fixed to 0.1 g/L and that the solution contained 0.1 wt% acetic acid in order to adjust the pH value to 3.4. The fit parameters are documented in Table S1 in the text below; Table S1. Parameters used to fit the SFG spectra shown in Figure S4. For assignments of these bands the reader is referred to the main text.

## References

[1] Lapitsky Y., Zahir T., Shoichet M.S. (2008). Modular biodegradable biomaterials from surfactant and polyelectrolyte mixtures. Biomacromolecules.

[2] Grant J., Lee H., Liu R.C.W., Allen C. (2008). Intermolecular interactions and morphology of aqueous polymer/surfactant mixtures containing cationic Chitosan and nonionic sorbitan esters. Biomacromolecules.

[3] Shi C., Zhu Y., Ran X., Wang M., Su Y., Cheng T. (2006). Therapeutic Potential of Chitosan and Its Derivatives in Regenerative Medicine. J. Surg. Res..

[4] Santini E., Jarek E., Ravera F., Liggieri L., Warszynski P., Krzan M. (2019). Surface properties and foamability of saponin and saponin-chitosan systems. Colloids Surf. B Biointerfaces.

[5] Oleszek W., Hamed A., Kjellin M., Johansson I. (2010). Saponin-Based Surfactants. Surfactants from Renewable Resources.

[6] Raafat D., Sahl H.G. (2009). Chitosan and its antimicrobial potential—A critical literature survey. Microbiol. Biotechnol..

[7] Wina E., Muetzel S., Becker K. (2005). The impact of saponins or saponin-containing plant materials on ruminant production—A review. J. Agric. Food Chem..

[8] Suzuki R., Ohno H., Murakami T., Shirataki Y. (2020). Improving quality control of yucca extracts used as food additives by screening antimicrobial activity using NMR metabolomics. J. Nat. Med..

[9] Guclu-Ustundag Ö., Mazza G. (2007). Saponins, Properties, applications and processing. Crit. Rev. Food Sci. Nutr..

[10] Nakabayashi T., Takakusagi Y., Iwabata K., Sakaguchi K. (2011). Foam fractionation of protein, Correlation of protein adsorption onto bubbles with a pH-induced conformational transition. Anal. Biochem..

[11] Jenkins K.J., Atwal A.S. (1994). Effects of dietary saponins on fecal bile acids and neutral sterols, and availability of vitamins A and E in the chick. J. Nutr. Biochem..

[12] Southon S., Wright A.J.A., Johnson I.T., Gee J.M., Price K., Fairweather-Tait S.J. (1988). The Effect of Saponins on Mineral Availability.

[13] Tamura Y., Miyakoshi M., Yamamoto M., Sakagami H. (2012). Application of Saponin-Containing Plants in Foods and Cosmetics. chapter 5. Alternative Medicine.

[14] Paul T.J., Taylor T.A., Rajendra Santosh A.B. (2020). The potential of saponin from Jamaica’s Blighia sapida (ackee) as a substitute for sodium lauryl sulphate in toothpaste. Med. Hypotheses.

[15] Cheeke P.R. (2000). Actual and potential applications of and saponins in human and animal nutrition. J. Anim. Sci..

[16] Devlieghere F., Vermeulen A., Debevere J. (2004). Chitosan, Antimicrobial activity, interactions with food components and applicability as a coating on fruit and vegetables. Food Microbiol..

[17] Kim I.Y., Seo S.J., Moon H.S., Yo M.-K., Park I.-Y., Kim B.-C., Cho C.-S. (2008). Chitosan and its derivatives for tissue engineering applications. Biotechnol. Adv..

[18] Dziza K., Santini E., Liggieri L., Jarek E., Krzan M., Fischer T.L., Ravera F. (2020). Interfacial Properties and Emulsification of Biocompatible Liquid-Liquid Systems. Coatings.

[19] Monteux C., Fuller G.G., Bergeron V. (2004). Shear and dilational surface rheology of oppositely charged polyelectrolyte/surfactant microgels adsorbed at the air-water interface. influence on foam stability. J. Phys. Chem. B.

[20] Golemanov K., Tcholakova S., Denkov N., Pelan E., Stoyanov S.D. (2012). Surface shear rheology of saponin adsorption layers. Langmuir.

[21] Glikman D., García Rey N., Richert M., Meister K., Braunschweig B. (2022). pH effects on the molecular structure and charging state of β-Escin biosurfactants at the air-water interface. J. Colloid Interface Sci..

[22] Kagiyama Y., Miyamae T. (2022). Interface Structure of Escin at Air-Water Interface probed by Sum Frequency Generation Spectroscopy. J. Raman. Spectrosc..

[23] Engelhardt K., Peukert W., Braunschweig B. (2014). Vibrational sum-frequency generation at protein modified air-water interfaces, Effects of molecular structure and surface charging. Curr. Opin. Colloid Interface Sci..

[24] Schulze-Zachau F., Braunschweig B. (2017). Structure of Polystyrenesulfonate/Surfactant Mixtures at Air-Water Interfaces and Their Role as Building Blocks for Macroscopic Foam. Langmuir.

[25] Richert M.E., García Rey N., Braunschweig B. (2018). Charge-Controlled Surface Properties of Native and Fluorophore-Labeled Bovine Serum Albumin at the Air-Water Interface. J. Phys. Chem. B.

[26] Engelhardt K., Weichsel U., Kraft E., Segets D., Peukert W., Braunschweig B. (2014). Mixed layers of β-lactoglobulin and SDS at air-water interfaces with tunable intermolecular interactions. J. Phys. Chem. B.

[27] Tyrode E., Hedberg J. (2012). A comparative study of the CD and CH stretching spectral regions of typical surfactants systems using VSFS, Orientation analysis of the terminal CH 3 and CD 3 groups. J. Phys. Chem. C.

[28] Lu R., Gan W., Wu B.H., Chen H., Wang H.F. (2004). Vibrational polarization spectroscopy of CH stretching modes of the methylene group at the vapor/liquid interfaces with sum frequency generation. J. Phys. Chem. B.

[29] Lu R., Gan W., Wu B.H., Zhang Z., Guo Y., Wang H.F. (2005). C-H stretching vibrations of methyl, methylene and methine groups at the vapor/Alcohol (n = 1–8) interfaces. J. Phys. Chem. B.

[30] Moghimipour E., Handali S. (2015). Saponin, Properties, Methods of Evaluation and Applications. Annu. Res. Rev. Biol..

[31] Scatena L.F., Brown M.G., Richmond G.L. (2001). Water at Hydrophobic Surfaces: Weak Hydrogen Bonding and Strong Orientation Effects. Science.

[32] Gonella G., Lütgebaucks C., de Beer A.G.F., Roke S. (2016). Second Harmonic and Sum-Frequency Generation from Aqueous Interfaces Is Modulated by Interference. J. Phys. Chem. C.

[33] Engelhardt K., Lexis M., Gochev G., Konnerth C., Miller R., Willenbacher N., Peukert W., Braunschweig B. (2013). pH Effects on the Molecular Structure of β-Lactoglobulin Modified Air–Water Interfaces and Its Impact on Foam Rheology. Langmuir.

[34] Guckeisen T., Hosseinpour S., Peukert W. (2019). Isoelectric Points of Proteins at the Air/Liquid Interface and in Solution. Langmuir.

[35] Richmond G.L. (2002). Molecular Bonding and Interactions at Aqueous Surfaces as Probed by Vibrational Sum Frequency Spectroscopy. Chem. Rev..

[36] Duffey K.C., Shih O., Wong N.L., Drisdell W.S., Saykally R.J., Cohen R.C. (2013). Evaporation kinetics of aqueous acetic acid droplets: Effects of soluble organic aerosol components on the mechanism of water evaporation. Phys. Chem. Chem. Phys..

[37] Schaefer J., Backus E.H.G., Nagata Y., Bonn M. (2016). Both Inter- and Intramolecular Coupling of O-H Groups Determine the Vibrational Response of the Water/Air Interface. J. Phys. Chem. Lett..

[38] Das S., Imoto S., Sun S., Nagata Y., Backus E.H.G., Bonn M. (2020). Nature of Excess Hydrated Proton at the Water-Air Interface. J. Am. Chem. Soc..

[39] Gragson D.E., Richmond G.L. (1998). Investigations of the Structure and Hydrogen Bonding of Water Molecules at Liquid Surfaces by Vibrational Sum Frequency Spectroscopy. J. Phys. Chem. B.

[40] Shen Y.R., Ostroverkhov V. (2006). Sum-frequency vibrational spectroscopy on water interfaces, Polar orientation of water molecules at interfaces. Chem. Rev..

[41] Sovago M., Campen R.K., Wurpel G.W.H., Müller M., Bakker H.J., Bonn M. (2008). Vibrational response of hydrogen-bonded interfacial water is dominated by intramolecular coupling. Phys. Rev. Lett..

[42] Ohto T., Backus E.H.G., Hsieh C.S., Sulpizi M., Bonn M., Nagata Y. (2015). Lipid Carbonyl Groups Terminate the Hydrogen Bond Network of Membrane-Bound Water. J. Phys. Chem. Lett..

[43] Nagata Y., Mukamel S. (2010). Vibrational sum-frequency generation spectroscopy at the water/lipid interface, Molecular dynamics simulation study. J. Am. Chem. Soc..

[44] Nojima Y., Suzuki Y., Yamaguchi S. (2017). Weakly Hydrogen-Bonded Water Inside Charged Lipid Monolayer Observed with Heterodyne-Detected Vibrational Sum Frequency Generation Spectroscopy. J. Phys. Chem. C.

[45] Mondal J.A., Nihonyanagi S., Yamaguchi S., Tahara T. (2012). Three distinct water structures at a zwitterionic lipid/water interface revealed by heterodyne-detected vibrational sum frequency generation. J. Am. Chem. Soc..

[46] Tielrooij K.J., Paparo D., Piatkowski L., Bakker H.J., Bonn M. (2009). Dielectric relaxation dynamics of water in model membranes probed by terahertz spectroscopy. Biophys. J..

[47] Zhao W., Moilanen D.E., Fenn E.E., Fayer M.D. (2008). Water at the surfaces of aligned phospholipid multibilayer model membranes probed with ultrafast vibrational spectroscopy. J. Am. Chem. Soc..

[48] Schnurbus M., Hardt M., Steinforth P., Carrascosa-Tejedor J., Winnall S., Gutfreund P., Schönhoff M., Campbell R.A., Braunschweig B. (2022). Responsive Material and Interfacial Properties through Remote Control of Polyelectrolyte–Surfactant Mixtures. ACS Appl. Mater. Interfaces.

[49] Schulze-Zachau F., Braunschweig B. (2019). CnTAB/polystyrene sulfonate mixtures at air-water interfaces, Effects of alkyl chain length on surface activity and charging state. Phys. Chem. Chem. Phys..

[50] Varga I., Campbell R.A. (2017). General physical description of the behavior of oppositely charged polyelectrolyte/surfactant mixtures at the air/water interface. Langmuir.

[51] Guzmán E., Llamas S., Maestro A., Fernandez-Pena L., Akanno A., Miller R., Ortega F., Rubio R.G. (2016). Polymer-surfactant systems in bulk and at fluid interfaces. Adv. Colloid Interface Sci..

[52] Richert M.E., Gochev G.G., Braunschweig B. (2019). Specific Ion Effects of Trivalent Cations on the Structure and Charging State of β-Lactoglobulin Adsorption Layers. Langmuir.

[53] Sthoer A., Tyrode E. (2019). Interactions of Na+ Cations with a Highly Charged Fatty Acid Langmuir Monolayer, Molecular Description of the Phase Transition. J. Phys. Chem. C.

[54] Hosseinpour S., Roeters S.J., Bonn M., Peukert W., Woutersen S., Weidner T. (2020). Structure and Dynamics of Interfacial Peptides and Proteins from Vibrational Sum-Frequency Generation Spectroscopy. Chem. Rev..

[55] Wojciechowski K. (2013). Surface activity of saponin from Quillaja bark at the air/water and oil/water interfaces. Colloids Surf. B Biointerfaces.

[56] Stanimirova R., Marinova K., Tcholakova S., Denkov N.D., Stoyanov S., Pelan E. (2011). Surface rheology of saponin adsorption layers. Langmuir.

[57] Mitra S., Dungan S.R. (1997). Micellar Properties of Quillaja Saponin. 1. Effects of Temperature, Salt, and pH on Solution Properties. J. Agric. Food Chem..

[58] Chen Z., Gracias D.H., Somorjai G.A. (1999). Sum frequency generation (SFG)—Surface vibrational spectroscopy studies of buried interfaces, Catalytic reaction intermediates on transition metal crystal surfaces at high reactant pressures, Polymer surface structures at the solid-gas and solid-liquid. Appl. Phys. B Lasers Opt..

[59] Chen Z., Shen Y.R., Somorjai G.A. (2002). Studies of polymer surfaces by sum frequency generation vibrational spectroscopy. Annu. Rev. Phys. Chem..

[60] Vidal F., Tadjeddine A. (2005). Sum-frequency generation spectroscopy of interfaces. Rep. Prog. Phys..

[61] Rey N.G., Dlott D.D. (2017). Studies of electrochemical interfaces by broadband sum frequency generation. J. Electroanal Chem..

[62] Chowdhury A.U., Muralidharan N., Daniel C., Amin R., Belharouak I. (2021). Probing the electrolyte/electrode interface with vibrational sum frequency generation spectroscopy, A review. J. Power Sources.

[63] Han H.L., Horowitz Y., Somorjai G.A. (2018). A Review on in Situ Sum Frequency Generation Vibrational Spectroscopy Studies of Liquid-Solid Interfaces in Electrochemical Systems. Encyclopedia of Interfacial Chemistry, Surface Science and Electrochemistry.

[64] Ding B., Chen Z. (2013). Sum Frequency Generation Vibrational Spectroscopy. Encycl. Biophys..

[65] Jubb A.M., Hua W., Allen H.C. (2012). Environmental chemistry at vapor/water interfaces, Insights from vibrational sum frequency generation spectroscopy. Annu. Rev. Phys. Chem..

[66] Nihonyanagi S., Mondal J.A., Yamaguchi S., Tahara T. (2013). Structure and dynamics of interfacial water studied by heterodyne-detected vibrational sum-frequency generation. Annu. Rev. Phys. Chem..

[67] Shen Y.R. (2012). Basic theory of surface sum-frequency generation. J. Phys. Chem. C.

[68] Wang H.F., Gan W., Lu R., Rao Y., Wu B.H. (2005). Quantitative spectral and orientational analysis in surface sum frequency generation vibrational spectroscopy (SFG-VS). Int. Rev. Phys. Chem..

[69] Bell G.R., Bain C.D., Ward R.N. (1996). Sum-frequency vibrational spectroscopy of soluble surfactants at the air/water interface. J. Chem. Soc.-Faraday Trans..

[70] Garciá Rey N., Weißenborn E., Schulze-Zachau F., Gochev G., Braunschweig B. (2019). Quantifying Double-Layer Potentials at Liquid-Gas Interfaces from Vibrational Sum-Frequency Generation. J. Phys. Chem. C.

[71] Tyrode E., Johnson C.M., Baldelli S., Leygraf C., Rutland M.W. (2005). A vibrational sum frequency spectroscopy study of the liquid-gas interface of acetic acid-water mixtures, 2. Orientation analysis. J. Phys. Chem. B.

[72] Tyrode E., Corkery R. (2018). Charging of Carboxylic Acid Monolayers with Monovalent Ions at Low Ionic Strengths, Molecular Insight Revealed by Vibrational Sum Frequency Spectroscopy. J. Phys. Chem. C.

[73] Hore D.K., Tyrode E. (2019). Probing Charged Aqueous Interfaces Near Critical Angles, Effect of Varying Coherence Length. J. Phys. Chem. C.

[74] Pool R.E., Versluis J., Backus E.H.G., Bonn M. (2011). Comparative study of direct and phase-specific vibrational sum-frequency generation spectroscopy, Advantages and limitations. J. Phys. Chem. B.

[75] Tian C.S., Shen Y.R. (2009). Structure and charging of hydrophobic material/water interfaces studied by phase-sensitive sum-frequency vibrational spectroscopy. Proc. Natl. Acad. Sci. USA..

[76] Sung J., Shen Y.R., Waychunas G.A. (2012). The interfacial structure of water/protonated α-Al_2_O_3_ ($11\bar{2}0$) as a function of pH. J. Phys. Condens. Matter..

[77] Ravera F., Loglio G., Kovalchuk V.I. (2010). Interfacial dilational rheology by oscillating bubble/drop methods. Curr. Opin. Colloid Interface Sci..

