# Supplementary material for: Surface Properties of Saponin—Chitosan Mixtures
Source: Molecules. 2022 Nov 3;27(21):7505. doi: 10.3390/molecules27217505 (PMC9658537; doi:10.3390/molecules27217505)
Supplement: Supplementary file 1 [file molecules-27-07505-s001.zip › molecules-1973778-supplementary.pdf]

Surface Properties of Saponin-Chitosan Mixtures

Marcel Krzan[1*], Natalia Garcia Rey[2], Ewelina Jarek[1], Agnieszka Czakaj[1], Eva Santini[3], Francesca Ravera[3], Libero Liggieri[3], Piotr Warszynski[1], Björn Braunschweig[2]

[1]*Jerzy Haber Institute of Catalysis and Surface Chemistry, Polish Academy of Sciences, Krakow, Poland*

[2] *Institute of Physical Chemistry and Center for Soft Nanoscience,* Westfälische Wilhelms-Universität Münster, Corrensstraße 28/30, 48149 Münster, Germany

[3] *Institute of Condensed Matter and Technologies for Energy, Consiglio Nazionale delle Ricerche Unit of Genoa, Genova, Italy*

** corresponding author: marcel.krzan@ikifp.edu.pl*

Table of contents

1. SFG spectra of saponin air-water interface 2

2. SFG spectra of chitosan air-water interface 3

3. Nonlinear least square fits to selected SFG spectra 3

4. References .... **Error! Bookmark not defined.**

1. SFG spectra of saponin air-water interface

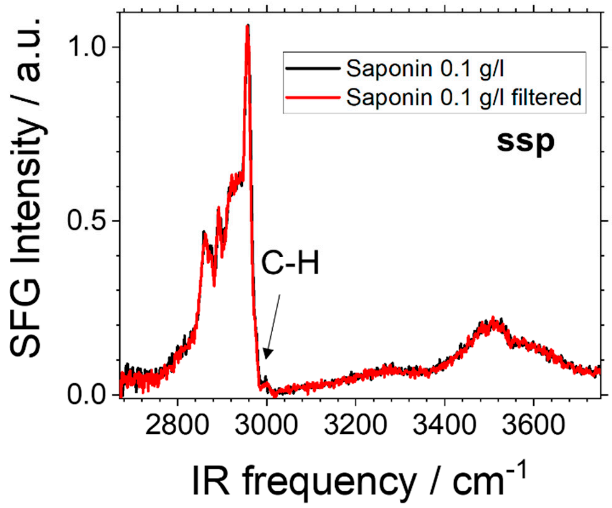

Figure S1. *Comparison of SFG spectra of air-water interfaces from filtered and unfiltered saponin solutions with a concentration of 0.1 g/L. The polarization of the SFG, VIS and IR beam is SSP. For the filtered sample the stock solution of saponin was filtered with a 0.1 µm filter.*

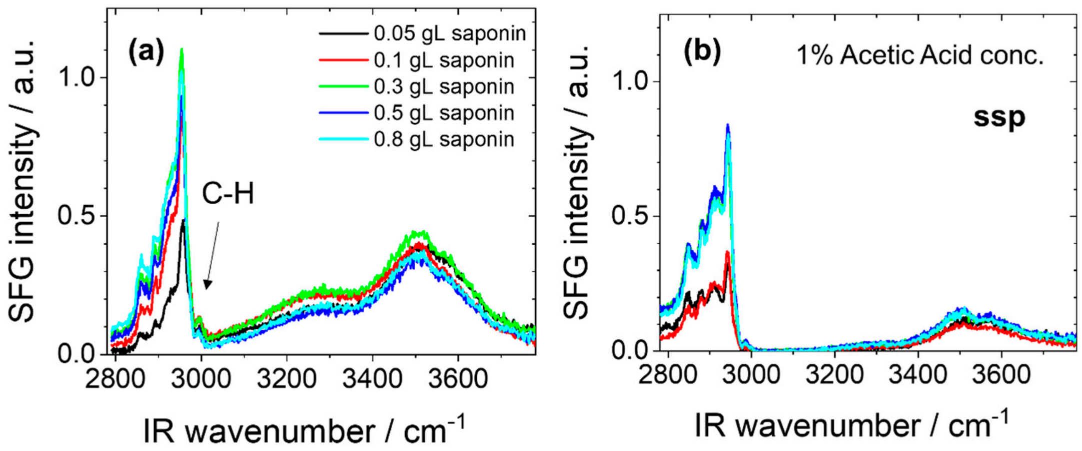

Figure S2*. SFG spectra of the pristine saponin air water interface. (a) study of the effect of the saponin concentration, pH 5.2 and (b) study of the addition of 1% acetic acid to the solutions (pH 2.85). The polarization of the SFG, VIS and IR beam was SSP. The pH of solutions with 1% of acetic acid becomes more acidic, pH 2.85, and the OH bands are highly affected by the double layer contribution, decreasing in SFG intensity due to the decrease in the net charge at the interface. The C-H bands also are affected by the tail of the broad OH stretching bands, as the SFG spectra are dependent on the interference between the bands and the relative phases. See references for detailed explanation [31-35].*

2. SFG spectra of chitosan air-water interface

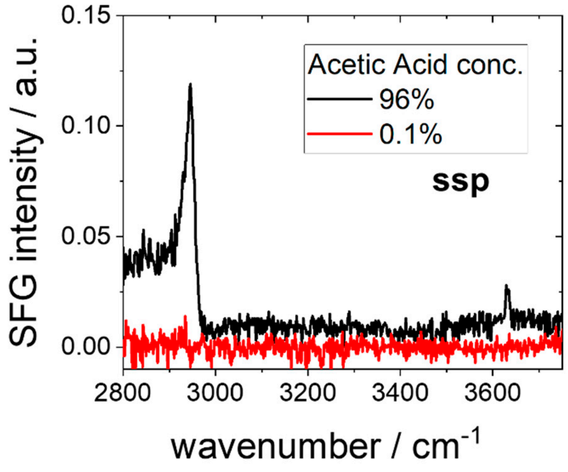

Figure S3. *Comparison of SFG spectra of 0.5 g/L chitosan air-water interface in 96% or 1% of acetic acid solution. The polarization of the SFG, VIS and IR beams is SSP, respectively.*

The SFG spectra of 0.5g/L chitosan in water and 1% acetic acid show non surface-active species. The chitosan molecules are not sufficiently present in the interfacial layer to detect it with SFG spectroscopy. We have include the SFG spectra of pure acetic acid (96% purity), were only one sharp band at 2945 cm^{-1} can be distinguished, attributed to the Fermi resonance of the $-CH_3$ of the acetic acid [36].

3. Nonlinear least square fits to selected SFG spectra

We have fitted the SFG intensity I_{SFG} using the following function:

$$I_{SFG} \propto \left|\chi_{eff}^{(2)}(\omega_{IR})\right|^2 = \left|\chi_{NR}^{(2)} + \sum_{k}^{5} \frac{A_k e^{-i\pi\varphi_k}}{\omega_{IR} - \omega_k + i\Gamma_k} + \sum_{q}^{3} \frac{A_q e^{-i\pi\varphi_q}}{\sqrt{2\pi\sigma_q}} V(\omega_q, \sigma_q, \Gamma_q)\right|^2 \qquad \text{S1.}$$

where we use the phase φ between the individually vibrational bands and the nonresonant background.

The fitting procedure was performed using a home-built MATLAB software code using the eq. S1. Where we used the Lorentzian terms for the narrow C-H vibrational bands as explained in the main text, while the broad O-H stretching bands (see also main text) that are centered at frequencies >3000 cm^{-1} where fitted with Voigt functions $V(\omega_q, \sigma_q, \Gamma_q)$ in order to account for inhomogeneous broadening. We point out that due to the high number of fit parameters needed to capture all the spectral details, fitting of the spectra is challenging and there might exists several sets of parameters that allow to fit the spectra with identical quality, which we did not explore in full detail. However, we decided to perform the fitting only for the lowest (0 g/L) and highest (0.5 g/L) chitosan concentration in order to show the change in relative phase of the water band close to 3200 cm^{-1} and the C-H band at 2994 cm^{-1} (Table 1). As discussed in detail in the main text, we link this change in relative phase to charge reversal at the interface.

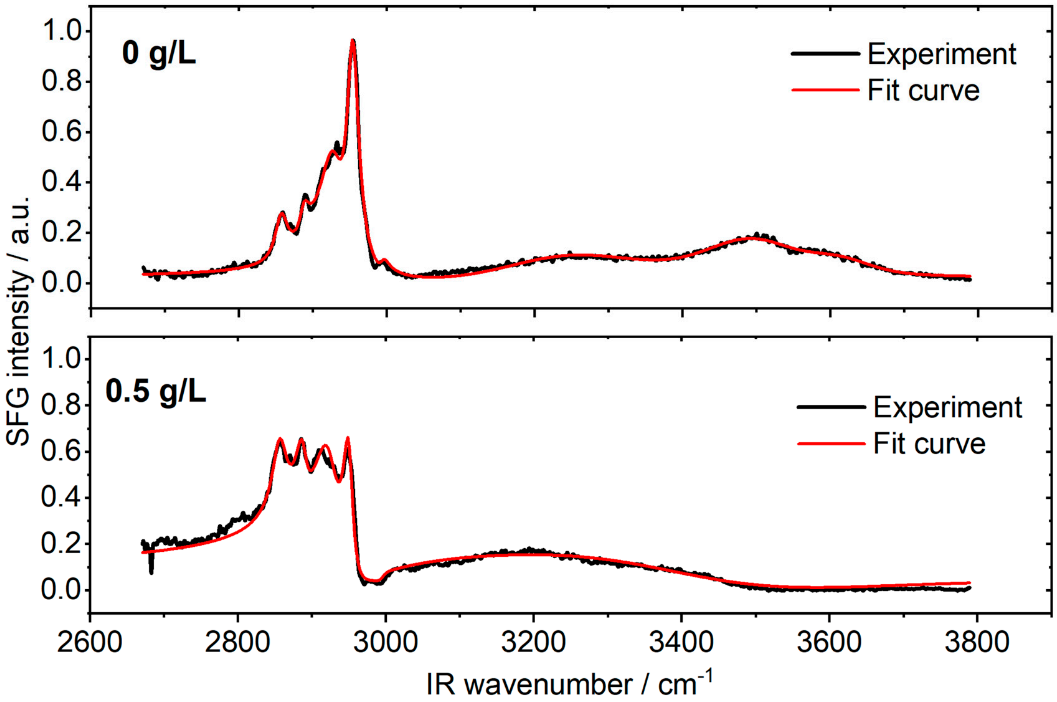

Figure S4. *Comparison of the fit curves (red lines) with the experimentally determined vibrational SFG spectra (black lines) of air-water interfaces modified by saponin-chitosan mixtures with chitosan concentrations of 0 and 0.5 g/L as indicated in the figure. Note that the saponin concentration in the aqueous solution was fixed to 0.1 g/l and that the solution contained 0.1 %wt acetic acid in order to adjust the pH value to 3.4. The fit parameters are documented in Table 1 in the text below.*

Table S1. *Parameters used to fit the SFG spectra shown in Figure S4. For assignments of these bands the reader is referred to the main text.*

		0 g/L	0.5 g/L
X_{NR}		0.15 ± 0.01	0.31 ± 0.17
d^+	A_k / a.u	0.21 ± 0.02	0.21 ± 0.02
	Γ_k / cm^{-1}	10	12
	φ / π	0.5	0.6
	ω_k / cm^{-1}	2857	2857
r^+	A_k / a.u	0.12 ± 0.01	0.16 ± 0.02
	Γ_k / cm^{-1}	8	9
	φ / π	0.5	0.8
	ω_k / cm^{-1}	2888	2888
d^+_{FR}	A_k / a.u	0.48 ± 0.02	0.39 ± 0.02
	Γ_k / cm^{-1}	20	20
	φ / π	1	1
	ω_k / cm^{-1}	2930	2930
r^- / r^+_{FR}	A_k / a.u	0.77 ± 0.01	0.51 ± 0.02
	Γ_k / cm^{-1}	10	8
	φ / π	1	1
	ω_k / cm^{-1}	2953	2951

		0 g/L	0.5 g/L
C-H	A_k / a.u	0.09 ± 0.01	0.35 ± 0.02
	Γ_k / cm^{-1}	7	8
	φ / π	1	0
	ω_k / cm^{-1}	2994	2994
O-H	A_q / a.u	16.8 ± 0.3	15.3 ± 0.4
	σ_q / cm^{-1}	82	150
	φ / π	1	1
	ω_q / cm^{-1}	3200	3150
O-H	A_q / a.u	12.0 ± 0.3	0.20 ± 0.02
	σ_q / cm^{-1}	58	127
	φ / π	0.6	1
	ω_q / cm^{-1}	3510	3364
O-H	A_q / a.u	3.8 ± 0.3	0.34 ± 0.1
	σ_q / cm^{-1}	40	92
	φ / π	0.4	1
	ω_q / cm^{-1}	3600	3550

References

1. Lapitsky, Y.; Zahir, T.; Shoichet, M.S. Modular biodegradable biomaterials from surfactant and polyelectrolyte mixtures. *Biomacromolecules* **2008**, *9*, 166–174. https://doi.org/10.1021/bm7009416.
2. Grant, J.; Lee, H.; Liu, R.C.W.; Allen, C. Intermolecular interactions and morphology of aqueous polymer/surfactant mixtures containing cationic Chitosan and nonionic sorbitan esters. *Biomacromolecules* **2008**, *9*, 2146–2152. https://doi.org/10.1021/bm800219m.
3. Shi, C.; Zhu, Y.; Ran, X.; Wang, M.; Su, Y.; Cheng, T. Therapeutic Potential of Chitosan and Its Derivatives in Regenerative Medicine. *J. Surg. Res.* **2006**, *133*, 185–192. https://doi.org/10.1016/j.jss.2005.12.013.
4. Santini, E.; Jarek, E.; Ravera, F.; Liggieri, L.; Warszynski, P.; Krzan, M. Surface properties and foamability of saponin and saponin-chitosan systems. *Colloids Surf. B Biointerfaces* **2019**, *181*, 198–206. https://doi.org/10.1016/j.colsurfb.2019.05.035.
5. Oleszek, W.; Hamed, A. Saponin-Based Surfactants. In *Surfactants from Renewable Resources*; Kjellin, M., Johansson, I., Eds.; Publisher: John Wiley & Sons, Ltd.: Hoboken, NJ, USA, 2010; pp. 239–249, ISBN 9780470760413. https://doi.org/10.1002/9780470686607.ch12.
6. Raafat, D.; Sahl, H.G. Chitosan and its antimicrobial potential—A critical literature survey. *Microbiol. Biotechnol.* **2009**, *2*, 186–201. https://doi.org/10.1111/j.1751-7915.2008.00080.x.
7. Wina, E.; Muetzel, S.; Becker, K. The impact of saponins or saponin-containing plant materials on ruminant production—A review. *J. Agric. Food Chem.* **2005**, *53*, 8093–8105. https://doi.org/10.1021/jf048053d.
8. Suzuki, R.; Ohno, H.; Murakami, T.; Shirataki, Y. Improving quality control of yucca extracts used as food additives by screening antimicrobial activity using NMR metabolomics. *J. Nat. Med.* **2020**, *74*, 306–310. https://doi.org/10.1007/s11418-019-01370-z.

9. Guclu-Ustundag, Ö.; Mazza, G. Saponins, Properties, applications and processing. *Crit. Rev. Food Sci. Nutr.* **2007**, *47*, 231–258. https://doi.org/10.1080/10408390600698197.
10. Nakabayashi, T.; Takakusagi, Y.; Iwabata, K.; Sakaguchi, K. Foam fractionation of protein, Correlation of protein adsorption onto bubbles with a pH-induced conformational transition. *Anal. Biochem*. **2011**, *419*, 173–179. https://doi.org/10.1016/j.ab.2011.08.019.
11. Jenkins, K.J.; Atwal, A.S. Effects of dietary saponins on fecal bile acids and neutral sterols, and availability of vitamins A and E in the chick. *J. Nutr. Biochem*. **1994**, *5*, 134–137. https://doi.org/10.1016/0955-2863(94)90084-1.
12. Southon, S.; Wright, A.J.A.; Johnson, I.T.; Gee, J.M.; Price, K.; Fairweather-Tait, S.J. *The Effect of Saponins on Mineral Availability*; AFRC Institute of Food Research: Colney Lane, Norwitch, UK, 1988; pp. 413–415.
13. Tamura, Y.; Miyakoshi, M.; Yamamoto, M. Application of Saponin-Containing Plants in Foods and Cosmetics. chapter 5. In *Alternative Medicine*; Sakagami, H., Ed.; IntechOpen: London, UK, 2012; ISBN 978-953-51-0903-7.
14. Paul, T.J.; Taylor, T.A.; Rajendra Santosh, A.B. The potential of saponin from Jamaica's Blighia sapida (ackee) as a substitute for sodium lauryl sulphate in toothpaste. *Med. Hypotheses* **2020**, *137*, 109555. https://doi.org/10.1016/j.mehy.2020.109555.
15. Cheeke, P.R. Actual and potential applications of and saponins in human and animal nutrition. *J. Anim. Sci*. **2000**, *77*, 1. https://doi.org/10.2527/jas2000.00218812007700es0009x.
16. Devlieghere, F.; Vermeulen, A.; Debevere, J. Chitosan, Antimicrobial activity, interactions with food components and applicability as a coating on fruit and vegetables. *Food Microbiol*. **2004**, *21*, 703–714. https://doi.org/10.1016/j.fm.2004.02.008.
17. Kim, I.Y.; Seo, S.J.; Moon, H.S.; Yo, M.-K.; Park, I.-Y.; Kim, B.-C.; Cho, C.-S. Chitosan and its derivatives for tissue engineering applications. *Biotechnol. Adv*. **2008**, *26*, 1–21. https://doi.org/10.1016/j.biotechadv.2007.07.009.
18. Dziza, K.; Santini, E.; Liggieri, L.; Jarek, E.; Krzan, M.; Fischer, T.L.; Ravera, F. Interfacial Properties and Emulsification of Biocompatible Liquid-Liquid Systems. *Coatings* **2020**, *10*, 397. https://doi.org/10.3390/coatings10040397.
19. Monteux, C.; Fuller, G.G.; Bergeron, V. Shear and dilational surface rheology of oppositely charged polyelectrolyte/surfactant microgels adsorbed at the air-water interface. influence on foam stability. *J. Phys. Chem. B* **2004**, *108*, 16473–16482. https://doi.org/10.1021/jp047462.
20. Golemanov, K.; Tcholakova, S.; Denkov, N.; Pelan, E.; Stoyanov, S.D. Surface shear rheology of saponin adsorption layers. *Langmuir* **2012**, *28*, 12071–12084. https://doi.org/10.1021/la302150j.
21. Glikman, D.; García Rey, N.; Richert, M.; Meister, K.; Braunschweig, B. pH effects on the molecular structure and charging state of β-Escin biosurfactants at the air-water interface. *J. Colloid Interface Sci*. **2022**, *607*, 1754–1761. https://doi.org/10.1016/j.jcis.2021.09.086.
22. Kagiyama, Y.; Miyamae, T. Interface Structure of Escin at Air-Water Interface probed by Sum Frequency Generation Spectroscopy. *J. Raman. Spectrosc*. **2022**, *53*, 1820. https://doi.org/10.1002/jrs.6418.
23. Engelhardt, K.; Peukert, W.; Braunschweig, B. Vibrational sum-frequency generation at protein modified air-water interfaces, Effects of molecular structure and surface charging. *Curr. Opin. Colloid Interface Sci*. **2014**, *19*, 207–215. https://doi.org/10.1016/j.cocis.2014.03.008.
24. Schulze-Zachau, F.; Braunschweig, B. Structure of Polystyrenesulfonate/Surfactant Mixtures at Air-Water Interfaces and Their Role as Building Blocks for Macroscopic Foam. *Langmuir* **2017**, *33*, 3499–3508. https://doi.org/10.1021/acs.langmuir.7b00400.
25. Richert, M.E.; García Rey, N.; Braunschweig, B. Charge-Controlled Surface Properties of Native and Fluorophore-Labeled Bovine Serum Albumin at the Air-Water Interface. *J. Phys. Chem. B* **2018**, *122*, 10377–10383. https://doi.org/10.1021/acs.jpcb.8b06481.
26. Engelhardt, K.; Weichsel, U.; Kraft, E.; Segets, D.; Peukert, W.; Braunschweig, B. Mixed layers of β-lactoglobulin and SDS at air-water interfaces with tunable intermolecular interactions. *J. Phys. Chem. B* **2014**, *118*, 4098–4105. https://doi.org/10.1021/jp501541q.
27. Tyrode, E.; Hedberg, J. A comparative study of the CD and CH stretching spectral regions of typical surfactants systems using VSFS, Orientation analysis of the terminal CH 3 and CD 3 groups. *J. Phys. Chem. C* **2012**, *116*, 1080–1091. https://doi.org/10.1021/jp210013g.
28. Lu, R.; Gan, W.; Wu, B.H.; Chen, H.; Wang, H.F. Vibrational polarization spectroscopy of CH stretching modes of the methylene group at the vapor/liquid interfaces with sum frequency generation. *J. Phys. Chem. B* **2004**, *108*, 7297–7306. https://doi.org/10.1021/jp036674o.
29. Lu, R.; Gan, W.; Wu, B.H.; Zhang, Z.; Guo, Y.; Wang, H.F. C-H stretching vibrations of methyl, methylene and methine groups at the vapor/Alcohol (n = 1–8) interfaces. *J. Phys. Chem. B* **2005**, *109*, 14118–14129. https://doi.org/10.1021/jp051565q.

30. Moghimipour, E.; Handali, S. Saponin, Properties, Methods of Evaluation and Applications. *Annu. Res. Rev. Biol.* **2015**, *5*, 207–220,. https://doi.org/10.9734/arrb/2015/11674.
31. Scatena, L.F.; Brown, M.G.; Richmond, G.L. Water at Hydrophobic Surfaces: Weak Hydrogen Bonding and Strong Orientation Effects. *Science* **2001**, *292*, 908–912. https://doi.org/10.1126/science.1059514.
32. Gonella, G.; Lütgebaucks, C.; de Beer, A.G.F.; Roke, S. Second Harmonic and Sum-Frequency Generation from Aqueous Interfaces Is Modulated by Interference. *J. Phys. Chem. C* **2016**, *120*, 9165–9173. https://doi.org/10.1021/acs.jpcc.5b12453.
33. Engelhardt, K.; Lexis, M.; Gochev, G.; Konnerth, C.; Miller, R.; Willenbacher, N.; Peukert, W.; Braunschweig, B. pH Effects on the Molecular Structure of β-Lactoglobulin Modified Air–Water Interfaces and Its Impact on Foam Rheology. *Langmuir* **2013**, *29*, 11646–11655. https://doi.org/10.1021/la402729g.
34. Guckeisen, T.; Hosseinpour, S.; Peukert, W. Isoelectric Points of Proteins at the Air/Liquid Interface and in Solution. *Langmuir* **2019**, *35*, 5004–5012. https://doi.org/10.1021/acs.langmuir.9b00311.
35. Richmond, G.L. Molecular Bonding and Interactions at Aqueous Surfaces as Probed by Vibrational Sum Frequency Spectroscopy. *Chem. Rev.* **2002**, *102*, 2693–2724. https://doi.org/10.1021/cr0006876.
36. Duffey, K.C.; Shih, O.; Wong, N.L.; Drisdell, W.S.; Saykally, R.J.; Cohen, R.C. Evaporation kinetics of aqueous acetic acid droplets: effects of soluble organic aerosol components on the mechanism of water evaporation. *Phys. Chem. Chem. Phys.* **2013**, *15*, 11634–11639. https://doi.org/10.1039/C3CP51148K.
37. Schaefer, J.; Backus, E.H.G.; Nagata, Y.; Bonn, M. Both Inter- and Intramolecular Coupling of O-H Groups Determine the Vibrational Response of the Water/Air Interface. *J. Phys. Chem. Lett.* **2016**, *7*, 4591–4595. https://doi.org/10.1021/acs.jpclett.6b02513.
38. Das, S.; Imoto, S.; Sun, S.; Nagata, Y.; Backus, E.H.G.; Bonn, M. Nature of Excess Hydrated Proton at the Water-Air Interface. *J. Am. Chem. Soc.* **2020**, *142*, 945–952. https://doi.org/10.1021/jacs.9b10807.
39. Gragson, D.E.; Richmond, G.L. Investigations of the Structure and Hydrogen Bonding of Water Molecules at Liquid Surfaces by Vibrational Sum Frequency Spectroscopy. *J. Phys. Chem. B* **1998**, *102*, 3847–3861. https://doi.org/10.1021/jp9806011.
40. Shen, Y.R.; Ostroverkhov, V. Sum-frequency vibrational spectroscopy on water interfaces, Polar orientation of water molecules at interfaces. *Chem. Rev.* **2006**, *106*, 1140–1154. https://doi.org/10.1021/cr040377d.
41. Sovago, M.; Campen, R.K.; Wurpel, G.W.H.; Müller, M.; Bakker, H.J.; Bonn, M. Vibrational response of hydrogen-bonded interfacial water is dominated by intramolecular coupling. *Phys. Rev. Lett.* **2008**, *100*, 173901. https://doi.org/10.1103/PhysRevLett.100.173901.
42. Ohto, T.; Backus, E.H.G.; Hsieh, C.S.; Sulpizi, M.; Bonn, M.; Nagata, Y. Lipid Carbonyl Groups Terminate the Hydrogen Bond Network of Membrane-Bound Water. *J. Phys. Chem. Lett.* **2015**, *6*, 4499–4503. https://doi.org/10.1021/acs.jpclett.5b02141.
43. Nagata, Y.; Mukamel, S. Vibrational sum-frequency generation spectroscopy at the water/lipid interface, Molecular dynamics simulation study. *J. Am. Chem. Soc.* **2010**, *132*, 6434–6442. https://doi.org/10.1021/ja100508n.
44. Nojima, Y.; Suzuki, Y.; Yamaguchi, S. Weakly Hydrogen-Bonded Water Inside Charged Lipid Monolayer Observed with Heterodyne-Detected Vibrational Sum Frequency Generation Spectroscopy. *J. Phys. Chem. C* **2017**, *121*, 2173–2180. https://doi.org/10.1021/acs.jpcc.6b09229.
45. Mondal, J.A.; Nihonyanagi, S.; Yamaguchi, S.; Tahara, T. Three distinct water structures at a zwitterionic lipid/water interface revealed by heterodyne-detected vibrational sum frequency generation. *J. Am. Chem. Soc.* **2012**, *134*, 7842–7850. https://doi.org/10.1021/ja300658h.
46. Tielrooij, K.J.; Paparo, D.; Piatkowski, L.; Bakker, H.J.; Bonn, M. Dielectric relaxation dynamics of water in model membranes probed by terahertz spectroscopy. *Biophys. J.* **2009**, *97*, 2484–2492. https://doi.org/10.1016/j.bpj.2009.08.024.
47. Zhao, W.; Moilanen, D.E.; Fenn, E.E.; Fayer, M.D. Water at the surfaces of aligned phospholipid multibilayer model membranes probed with ultrafast vibrational spectroscopy. *J. Am. Chem. Soc.* **2008**, *130*, 13927–13937. https://doi.org/10.1021/ja803252y.
48. Schnurbus, M.; Hardt, M.; Steinforth, P.; Carrascosa-Tejedor, J.; Winnall, S.; Gutfreund, P.; Schönhoff, M.; Campbell, R.A.; Braunschweig, B. Responsive Material and Interfacial Properties through Remote Control of Polyelectrolyte–Surfactant Mixtures. *ACS Appl. Mater Interfaces* **2022**, *14*, 4656–4667. https://doi.org/10.1021/acsami.1c18934.
49. Schulze-Zachau, F.; Braunschweig, B. CnTAB/polystyrene sulfonate mixtures at air-water interfaces, Effects of alkyl chain length on surface activity and charging state. *Phys. Chem. Chem. Phys.* **2019**, *21*, 7847–7856. https://doi.org/10.1039/c9cp01107b.

50. Varga, I.; Campbell, R.A. General physical description of the behavior of oppositely charged polyelectrolyte/surfactant mixtures at the air/water interface. *Langmuir* **2017**, *33*, 5915–5924. https://doi.org/10.1021/acs.langmuir.7b01288.
51. Guzmán, E.; Llamas, S.; Maestro, A.; Fernandez-Pena, L.; Akanno, A.; Miller, R.; Ortega, F.; Rubio, R.G. Polymer-surfactant systems in bulk and at fluid interfaces. *Adv. Colloid Interface Sci*. **2016**, *233*, 38–64. https://doi.org/10.1016/j.cis.2015.11.001.
52. Richert, M.E.; Gochev, G.G.; Braunschweig, B. Specific Ion Effects of Trivalent Cations on the Structure and Charging State of β-Lactoglobulin Adsorption Layers. *Langmuir* **2019**, *35*, 11299–11307. https://doi.org/10.1021/acs.langmuir.9b01803.
53. Sthoer, A.; Tyrode, E. Interactions of Na+ Cations with a Highly Charged Fatty Acid Langmuir Monolayer, Molecular Description of the Phase Transition. *J. Phys. Chem. C* **2019**, *123*, 23037–23048. https://doi.org/10.1021/acs.jpcc.9b06435.
54. Hosseinpour, S.; Roeters, S.J.; Bonn, M.; Peukert, W.; Woutersen, S.; Weidner, T. Structure and Dynamics of Interfacial Peptides and Proteins from Vibrational Sum-Frequency Generation Spectroscopy. *Chem. Rev*. **2020**, *120*, 3420–3465. https://doi.org/10.1021/acs.chemrev.9b00410.
55. Wojciechowski, K. Surface activity of saponin from Quillaja bark at the air/water and oil/water interfaces. *Colloids Surf. B Biointerfaces* **2013**, *108*, 95–102. https://doi.org/10.1016/j.colsurfb.2013.02.008.
56. Stanimirova, R.; Marinova, K.; Tcholakova, S.; Denkov, N.D.; Stoyanov, S.; Pelan, E. Surface rheology of saponin adsorption layers. *Langmuir* **2011**, *27*, 12486–12498. https://doi.org/10.1021/la202860u.
57. Mitra, S.; Dungan, S.R. Micellar Properties of Quillaja Saponin. 1. Effects of Temperature, Salt, and pH on Solution Properties. *J. Agric. Food Chem*. **1997**, *45*, 1587–1595. https://doi.org/10.1021/jf960349z.
58. Chen, Z.; Gracias, D.H.; Somorjai, G.A. Sum frequency generation (SFG)—Surface vibrational spectroscopy studies of buried interfaces, Catalytic reaction intermediates on transition metal crystal surfaces at high reactant pressures, Polymer surface structures at the solid-gas and solid-liquid . *Appl. Phys. B Lasers Opt*. **1999**, *68*, 549–557. https://doi.org/10.1007/s003400050664.
59. Chen, Z.; Shen, Y.R.; Somorjai, G.A. Studies of polymer surfaces by sum frequency generation vibrational spectroscopy. *Annu. Rev. Phys. Chem*. **2002**, *53*, 437–465. https://doi.org/10.1146/annurev.physchem.53.091801.115126.
60. Vidal, F.; Tadjeddine, A. Sum-frequency generation spectroscopy of interfaces. *Rep. Prog. Phys*. **2005**, *68*, 1095–1127. https://doi.org/10.1088/0034-4885/68/5/R03.
61. Rey, N.G.; Dlott, D.D. Studies of electrochemical interfaces by broadband sum frequency generation. *J. Electroanal Chem*. **2017**, *800*, 114–125. https://doi.org/10.1016/j.jelechem.2016.12.023.
62. Chowdhury, A.U.; Muralidharan, N.; Daniel, C.; Amin, R.; Belharouak, I. Probing the electrolyte/electrode interface with vibrational sum frequency generation spectroscopy, A review. *J. Power Sources* **2021**, *506*, 230173. https://doi.org/10.1016/j.jpowsour.2021.230173.
63. Han, H.L.; Horowitz, Y.; Somorjai, G.A. A Review on in Situ Sum Frequency Generation Vibrational Spectroscopy Studies of Liquid-Solid Interfaces in Electrochemical Systems. In *Encyclopedia of Interfacial Chemistry, Surface Science and Electrochemistry*; Elsevier: Amsterdam, The Netherlands, 2018, pp. 1–12. https://doi.org/10.1016/B978-0-12-409547-2.13730-8.
64. Ding, B.; Chen, Z. Sum Frequency Generation Vibrational Spectroscopy. *Encycl. Biophys*. **2013**, *19*, 2512–2516. https://doi.org/10.1007/978-3-642-16712-6_142.
65. Jubb, A.M.; Hua, W.; Allen, H.C. Environmental chemistry at vapor/water interfaces, Insights from vibrational sum frequency generation spectroscopy. *Annu. Rev. Phys. Chem*. **2012**, *63*, 109–130. https://doi.org/10.1146/annurev-physchem-032511-143811.
66. Nihonyanagi, S.; Mondal, J.A.; Yamaguchi, S.; Tahara, T. Structure and dynamics of interfacial water studied by heterodyne-detected vibrational sum-frequency generation. *Annu. Rev. Phys. Chem*. **2013**, *64*, 579–603. https://doi.org/10.1146/annurev-physchem-040412-110138.
67. Shen, Y.R. Basic theory of surface sum-frequency generation. *J. Phys. Chem. C* **2012**, *116*, 15505–15509. https://doi.org/10.1021/jp305539v.
68. Wang, H.F.; Gan, W.; Lu, R.; Rao, Y.; Wu, B.H. Quantitative spectral and orientational analysis in surface sum frequency generation vibrational spectroscopy (SFG-VS). *Int. Rev. Phys. Chem*. **2005**, *24*, 191–256. https://doi.org/10.1080/01442350500225894.
69. Bell, G.R.; Bain, C.D.; Ward, R.N. Sum-frequency vibrational spectroscopy of soluble surfactants at the air/water interface. *J. Chem. Soc.-Faraday Trans*. **1996**, *92*, 515–523. https://doi.org/10.1039/ft9969200515.

70. Garciá Rey, N.; Weißenborn, E.; Schulze-Zachau, F.; Gochev, G.; Braunschweig, B. Quantifying Double-Layer Potentials at Liquid-Gas Interfaces from Vibrational Sum-Frequency Generation. *J. Phys. Chem. C* **2019**, *123*, 1279–1286. https://doi.org/10.1021/acs.jpcc.8b10097.
71. Tyrode, E.; Johnson, C.M.; Baldelli, S.; Leygraf, C.; Rutland, M.W. A vibrational sum frequency spectroscopy study of the liquid-gas interface of acetic acid-water mixtures, 2. Orientation analysis. *J. Phys. Chem. B* **2005**, *109*, 329–341. https://doi.org/10.1021/jp047337y.
72. Tyrode, E.; Corkery, R. Charging of Carboxylic Acid Monolayers with Monovalent Ions at Low Ionic Strengths, Molecular Insight Revealed by Vibrational Sum Frequency Spectroscopy. *J. Phys. Chem. C* **2018**, *122*, 28775–28786. https://doi.org/10.1021/acs.jpcc.8b09505.
73. Hore, D.K.; Tyrode, E. Probing Charged Aqueous Interfaces Near Critical Angles, Effect of Varying Coherence Length. *J. Phys. Chem. C* **2019**, *123*, 16911–16920. https://doi.org/10.1021/acs.jpcc.9b05256.
74. Pool, R.E.; Versluis, J.; Backus, E.H.G.; Bonn, M. Comparative study of direct and phase-specific vibrational sum-frequency generation spectroscopy, Advantages and limitations. *J. Phys. Chem. B* **2011**, *115*, 15362–15369. https://doi.org/10.1021/jp2079023.
75. Tian, C.S.; Shen, Y.R. Structure and charging of hydrophobic material/water interfaces studied by phase-sensitive sum-frequency vibrational spectroscopy. *Proc. Natl. Acad. Sci. USA*. **2009**, *106*, 15148–15153. https://doi.org/10.1073/pnas.0901480106.
76. Sung, J.; Shen, Y.R.; Waychunas, G.A. The interfacial structure of water/protonated α-Al 2O 3 (11$\bar{2}$0) as a function of pH. *J. Phys. Condens. Matter*. **2012**, *24*, 124101. https://doi.org/10.1088/0953-8984/24/12/124101.
77. Ravera, F.; Loglio, G.; Kovalchuk, V.I. Interfacial dilational rheology by oscillating bubble/drop methods. *Curr. Opin. Colloid Interface Sci*. **2010**, *15*, 217–228. https://doi.org/10.1016/j.cocis.2010.04.001.
